# A comparison of single and intersectional social identities associated with discrimination and mental health service use: data from the 2014 Adult Psychiatric Morbidity Survey in England

**DOI:** 10.1007/s00127-022-02259-1

**Published:** 2022-03-07

**Authors:** Rebecca D. Rhead, Charlotte Woodhead, Gargie Ahmad, Jayati Das-Munshi, Sally McManus, Stephani L. Hatch

**Affiliations:** 1 Department of Psychological Medicine, Institute of Psychiatry, Psychology and Neuroscience, King’s College London, 3rdfloor E3.14, London, SE5 8AB UK; 2 ESRC Centre for Society and Mental Health, King’s College London, London, UK; 3 South London and Maudsley (SLaM) NHS Trust, London, UK; 4 National Centre for Social Research, London, UK; 5 Violence and Society Centre, City, University of London, London, UK

**Keywords:** Inequalities, Discrimination, Mental health, Latent class analysis, Intersectionality

## Abstract

Inequities in mental health service use (MHSU) and treatment are influenced by social stratification processes linked to socially contextualised interactions between individuals, organisations and institutions. These complex relations underpin observed inequities and their experience by people at the intersections of social statuses. Discrimination is one important mechanism influencing such differences. We compared inequities in MHSU/treatment through single and intersectional status analyses, accounting for need. We assessed whether past-year discrimination differentially influences MHSU/treatment across single and intersecting statuses. Data came from a population survey (collected 2014–2015) nationally representative of English households (*N* = 7546). We used a theory and datadriven approach (latent class analysis) which identified five intersectional groups in the population comprising common combinations of social statuses. Single status analyses identified characteristics associated with MHSU/treatment (being a sexual minority (adjusted odds ratio (AOR) 1.65 95% CI:1.09-2.50), female (AOR 1.71, 95% CI:1.45–2.02), economically inactive (AOR 2.02, 95% CI:1.05–3.90), in the most deprived quintile (AOR 1.33, 95% CI:1.02–1.74), and Black (AOR 0.36 95% CI:0.20–0.66)). Intersectional analyses detected patterns not apparent from single status analyses. Compared to the most privileged group (“White British, highly educated, employed, high social class”), “Retired White British” had greater odds of MHSU/treatment (AOR 1.88, 95% CI:1.53-2.32) while “Employed migrants” had lower odds (AOR 0.39, 95% CI:0.27–0.55). Past-year discrimination was associated with certain disadvantaged social statuses and greater MHSU/treatment but—except for sexual minorities—adjusting for discrimination had little influence using either analytic approach. Observing patterns only by single social statuses masks potentially unanticipated and contextually varying inequities. The latent class approach offers policy-relevant insights into patterns and mechanisms of inequity but may mask other key intersectional patterns by statuses less common or under represented in surveys (e.g. UK-born ethnic minority groups). We propose multiple, context-relevant, theory-driven approaches to intersectional understanding of mental health inequalities.

**Supplementary Information:**

The online version contains supplementary material available at 10.1007/s00127-022-02259-1.

## Introduction

Stress process theory describes how social stratification systems lead to inequalities in mental health through accumulative lifetime exposure to adversities and disparities in access to social, cultural and material resources [47]. Such hierarchical categorisations (e.g., on the basis of race/ethnicity, gender or social class) are emergent from, maintained by, and transformed through interactions between individuals, organisations and institutions within and across social contexts [36]. However, they relate to stress and mental health in complex ways [37], reflected in the differences in experience across and at the intersections of various statuses and contexts (e.g., social class). This is in addition to the inter- and intra-personal processes linked to social identity, meaning construction, and reflexive deliberation [36, 57]. To understand the mechanisms through which observed patterns of inequality are produced and to inform interventions, sociological studies of mental health inequ(al)ities should incorporate and explore this complexity.

One such inequity pertains to the distribution of support for commonly occurring mental disorders, such as anxiety and depression. Despite being a major cause of disability, internationally, there is a large treatment gap and disparities across population groups [29, 39]. Studies have identified several social status characteristics associated with inequitable mental health service use [18, 38, 39] and indicate structural and subjective barriers arising in patient, provider and healthcare system interactions [52, 58].

Seeking help or maintaining service engagement is a socially negotiated process influenced by biases and discrimination based on social statuses, such as race/ethnicity, religion, age, gender, socio-economic status (SES), sexual orientation, and migration status [30, 33]. However, such systems of stratification are likely to shape access to and uptake of mental health support in complex and contextually specific ways. For example, among women but not men, Pakistani and Bangladeshi ethnic groups in England are less likely than White groups to have used mental health services [27]; and, lower rates of primary care depression screening have been reported among Black than White non-Latinx patients in the United States—with an elevated discrepancy among females [25]. Nonetheless, there is little understanding about whether and why people occupying multiple advantaged or disadvantaged statuses differentially access mental health treatment or services.

Intersectionality theory [14] emphasises the simultaneity of multiple social statuses (gender, race/ethnicity, social class, etc.) by recognising people’s experiences of being at a junction of more than one social status or position, each, respectively, shaped by social power. It is therefore inadequate to examine statuses in isolation [11]. People’s multiple statuses may include a mix of privileged and disadvantaged social categories [36] which influence each other, adding further complexity to people’s experiences embedded in different systems of stratification [14, 64]. This is supported by studies finding that patterns of inequalities in mental health differ when examining social statuses singly or intersectionally [22, 24]. Moreover, there may be contextual differences in patterns of inequality in mental health treatment [17]. English-based research from South East London has found that, compared to privileged groups, groups characterised by multiply disadvantaged or a mix of advantaged and disadvantaged positions were *more* likely to be in receipt of treatment for mental health reasons after accounting for need [21]. The extent to which this is reflected in the wider national setting is unknown.

### Moving beyond describing inequalities

Quantitative studies assessing intersectionality focus on two main approaches: descriptively examining outcomes by generating categories representing multiple statuses, or regressing an outcome on multiple social status markers and their interactions [5, 6]. While descriptive approaches are important for identifying intersectional differences in outcomes, analyses incorporating markers of modifiable causal mechanisms are also important to monitor and tackle such inequities [6]. This ‘analytical intersectionality’ should also permit examination of differences in mechanisms underpinning inequities across intersectional status groups, such that neither outcomes nor underlying processes are assumed additive. Here, underlying processes are considered ‘causal’, to the extent that the influences of multiple social advantages or disadvantages on outcomes occur through, or are mediated by, other social processes.

Social stressors, such as stigma and discrimination, are important mechanisms underpinning status-based inequities in mental health treatment and support [6, 7, 13]. Such stressors are grounded in wider social inequalities experienced as both acute life events and chronic hassles. Discrimination—alone and particularly in conjunction with other stressors—is widely demonstrated to elicit psychological and physiological stress responses that impair health [32, 50, 56, 63, 65]. As a stressor, discrimination has adverse effects linked to social devaluation and reduced mastery, eroding psychosocial resources protective against further discriminatory experiences and stress [36, 48, 49]. Discrimination may therefore predict not only greater need for support but also elevated service use [21]. In contrast, experiencing and anticipating discrimination may also inhibit help-seeking by increasing mistrust and fear, affecting service use discontinuation and deterring future service engagement, thus also impacting upon outcomes [26, 56]. Moreover, the experience and impact of discrimination on mental health and service use are also likely to differ within, at the intersections of, and across social statuses [32, 60, 64].

This study builds on previous UK-based work in an urban diverse setting in Southeast London which examined associations between discrimination and health service use for mental health reasons. Adjusting for intersecting social statuses and need, they found that anticipated discrimination predicted higher levels of use supporting its role as a stressor [21]. This study utilises a similar approach using national population-based data representative of households in England to examine mental health service use (MHSU) and treatment at the intersections of multiple advantaged and disadvantaged social statuses. It examines whether discrimination has a differential influence on MHSU/treatment across different intersecting social statuses after accounting for markers of need. Specifically, we address the following hypotheses:

H1: People with single and multiple disadvantaged social statuses will report greater discrimination and greater MHSU/treatment than more advantaged groups after accounting for need.
H2: Patterns of discrimination and MHSU/treatment will differ when considering single, compared to multiple social statuses.
H3: Accounting for discrimination will partially attenuate associations between social status and MHSU/treatment.
H4: Discrimination will have a greater influence on MHSU/treatment for people occupying multiply disadvantaged social statuses than more advantaged groups, or groups comprising a mix of advantaged and disadvantaged social statuses.

## Methods

### Data sources

#### Adult psychiatric morbidity survey

The 2014 Adult Psychiatric Morbidity Survey (APMS) provides data collected between 2014 and 2015 on the prevalence of psychiatric disorders in the English adult population [39]. The sample (*N* = 7546) was designed to be representative of the population aged 16 years and above living in private households in England. APMS incorporated assessment of common mental disorders, substance misuse and less prevalent psychiatric disorders, such as psychosis. It also collected data on demographic and socio-economic characteristics, discrimination, stressful life events, social support and health service use. Detailed survey profile information is available [40].

### Measures

#### Mental health service use and treatment

APMS permits identification of people receiving any treatment for mental ill health, including any current use of medication (excluding medication solely for dementia, dependence or epilepsy) and any current counselling or therapy. MHSU is indicated by any inpatient or outpatient use of hospital services for mental ill health in the past quarter, or any past-year contact with a GP about being anxious or depressed or a mental, nervous or emotional problem.

#### Need

We examined variables identified in a recent systematic review [54] as key need factors predicting MHSU for common mental disorders. Severity of symptoms was assessed using the Revised Clinical Interview Schedule (CIS-R) [31]. This structured interview asks about 14 symptom domains: fatigue, sleep problems, irritability, worry, depression, depressive ideas, anxiety, obsessions, subjective memory and concentration, somatic symptoms, compulsions, phobias, physical health worries and panic. We grouped scores as 0–5 (little or no symptoms), 6–11 (sub-threshold), 12–17 (symptoms warranting primary care recognition), or 18 + (symptoms very likely to warrant intervention) [31, 39]. We also included a binary marker of self-reported general health (fair/poor vs good/very good/excellent) and of any physical illness conditions in the past 12 months. Finally, we included a measure of social functioning using the eight-item Social Functioning Questionnaire (SFQ, [61] which assesses ratings of performance and stress related to domains, such as work, household tasks, financial matters, relationships and spare time. We developed a binary variable, where the median (a score of 14) was used to identify ‘better’ (< 14) and ‘worse’ (14 +) social functioning.

#### Intersectional social status indicators

APMS collects data on ethnicity aligned with the UK census, where participants are asked to choose from eighteen categories across five broad sections to best describe race/ethnic group. Each section contains an ‘Any Other’ category to permit individuals for whom existing options do not apply to self-complete. We distinguished five broad groups due to small sample sizes in the ethnic minority categories, as in previous work with this dataset [39]: White British, White Other, Black/African/Caribbean/Black British, Asian/Asian British, Mixed/Multiple/Other.

Migration status was derived from country of birth, distinguishing migrants (non-UK-born) and non-migrants (UK-born). Gender was available only as a binary variable (male or female). Sexual orientation was included, although was only asked of participants aged under 65 years. We distinguished people identifying as heterosexual or straight from those identifying as gay or lesbian, bisexual, or other (‘sexual minority’), and those unclassified (participants aged 65 + who are not asked to disclose their sexual orientation).

Socio-economic indicators included educational qualification attainment (none, GCSE/vocational-level, A-level, degree level); employment status (employed, unemployed, and three economically inactive groups: student, retired, and other—including sick/disabled and looking after family home); and social class, measured through the National Statistics Socio-economic Classification (NS-SEC) which ordinally categorises socio-economic groups based on employment relations and occupational conditions (Office for National Statistics (ONS) 2019). We used the four-category NS-SEC version, distinguishing managerial/professional, intermediate, routine/manual, or not worked recently.

#### Assessing intersectionality

Following from previous work [10, 21, 22], we used latent class analysis (LCA), a data-driven method to define intersectional groups with similar profiles according to salient characteristics of social stratification: socio-economic status (education, employment status, social class), ethnicity, migration status, gender, and sexual orientation. In LCA, individuals are categorised based on conditional probabilities such that members of each class have similar patterns of responses to the variables included. Previous intersectionality studies have used the traditional ‘classify-analyse method’ to examine associations between latent class membership and distal outcomes (participants are assigned to a latent class, these data are then exported and analysed separately). However, this approach is now contraindicated [44] due to risk of classification error tending to produce attenuated estimates (biased towards zero) and standard errors for the effect of latent class membership on distal outcomes of interest [4, 9].

In our examination of distal outcomes, we therefore used a three-step approach which can account for classification error [8]. This is achieved by identifying the best fitting LCA model and saving the posterior probabilities and modal class assignment for that model. Classification errors for individuals are then computed, and the inverse logits of those individual-level error rates are used as weights. The reweighted data are then used as observed data to estimate associations with distal outcomes of interest [2, 3].

#### Confounders

Potential confounders of the association between social status measures and MHSU, between social status measures and discrimination, and between discrimination and MHSU included age (recorded in APMS in 10 year age bands (16–24, 25–34, 35–44, 45–54, 55–64, 65–74, 75 +), marital status (single, married/civil partnered/cohabiting, separated/divorced/widowed), a binary variable to indicate urban/rural residence, and area-level deprivation quintile (Index of Multiple Deprivation, IMD) [23].

#### Discrimination

APMS asked participants if they had been treated unfairly in the past year because of age, sex, ethnicity, religion, sexual orientation, physical health, or mental health. To explore whether discrimination attributable to *multiple* social statuses was related to mental health service use, we developed a binary indicator of past-year unfair treatment for any reason (no/yes) rather than restricting analyses to a single attribution [6].

### Statistical analyses

Using MPlus [42], optimal latent class models were developed for men and women. We selected the optimal number of classes using a series of goodness of fit (GoF) statistics: Akaike's information criteria (AIC) [1], sample-size-adjusted Bayesian information criteria (SSABIC) [55], and the Lo–Mendell–Rubin-Adjusted likelihood ratio test (LMRA–LRT) [34]. Lower values for these indicate a better fit. Entropy measures accuracy of classification for an individual participant with higher values indicative of better classification [53]. To distinguish between classes with similar GoF statistics, the LMRA–LRT *p* values were assessed, with significance indicative of good fit. Finally, parsimony and interpretability were considered in deciding the optimal number of classes [43].

Next, logistic regression analyses were used to explore associations between (i) single social status characteristics and any past-year discrimination (considered to be a mental health stressor, [21], and (ii) intersectional latent class groups and any past-year discrimination. We present unadjusted and adjusted odds ratios (OR/AOR) with 95% confidence intervals (CI). Adjustments were made for all socio-demographic/economic characteristics: age, marital status, urban/rural residence, area-level deprivation.

**Fig. 1:**
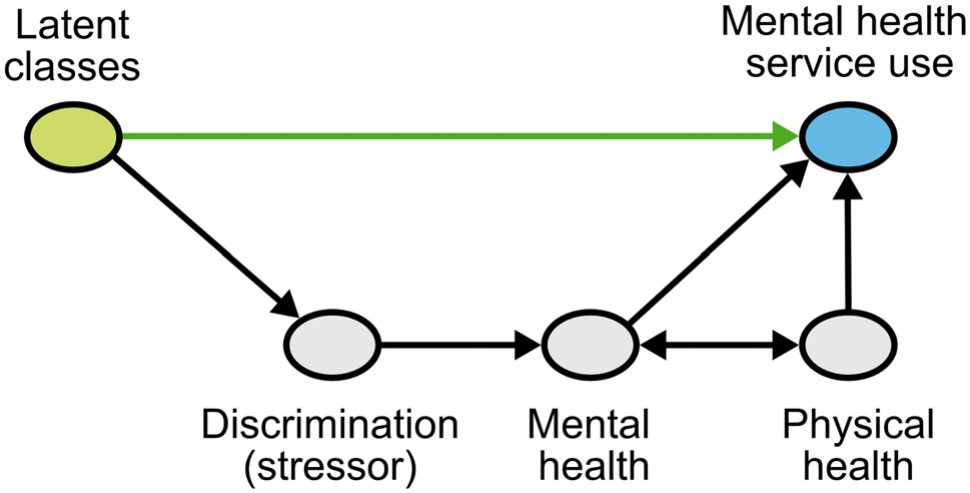
Directed acyclic graph to illustration the relationships modelled in our analysis

Finally, a series of multivariable logistic regression models were estimated to examine associations between (i) single social statuses and MHSU/treatment and (ii) intersectional status (latent classes) and MHSU/treatment (incorporating the three-step approach discussed above). OR/AORs and 95% CIs for the following models are presented, in which additional variables were sequentially added in the order described: (1) unadjusted; and adjusted for: (2) socio-demographic/economic confounders (including as appropriate: age, gender, marital status, urban/rural residence, area-level deprivation, ethnicity, social class, employment status, migration status, sexual orientation, educational attainment); (3) past-year discrimination; (4) CIS-R symptom severity and (5) self-reported general health, physical illness and social functioning (see Fig. 1).

Acknowledging the limitations of mediation analysis using cross-sectional data, evidence for mediation by discrimination was putatively indicated if discrimination was associated with single or intersectional social statuses and with MHSU/treatment, and adjustments for discrimination attenuated associations between status and MHSU/treatment (Kenny n.d.). Analyses accounted for study design including strata, clustering and survey weights.

## Results

### Intersectional social statuses—Latent Class Analysis (LCA)

Initially, five-class solutions were selected as optimal for both men and women (see Supplementary file). We found almost identical classes and similar patterns of findings for both samples. However, stratifying by gender led to low numbers for some classes. This was particularly noticeable for the male sample which is both smaller than the female sample and has lower engagement with mental health services. We therefore decided to conduct analyses using the full sample and including gender as an indicator for the latent class model. We found the same class composition overall as in the separate models for men and women.

The classes in our final model (Table 1) were characterised as predominantly: (1) Employed White British, educated to degree level and belonging to the managerial/professional NS-SEC category (designated most privileged) (23.7% of the sample). (2) Employed White British, educated below degree level and belonging to the routine/manual NS-SEC category, this class contains the most men (26.4%); (3) Primarily non-working migrants, of varied ethnicity and education (4.6%), this class contains the most women; (4) Employed migrants, of varied ethnicity, social class and education level (6.9%), this class contains more highly educated and economically active participants than LCA3; and (5) Retired White British (38.3%). There was not much variation between classes in conditional probabilities of being either female or a sexual minority.

**Table 1:** Item probabilities for Latent Class Analysis (LCA) model

Indicators	LC1, *n* = 1791	LC2, *n* = 1994	LC3, *n* = 348	LC4, *n* = 524	LC5, *n* = 2889
Ethnicity					
White British	0.913	0.961	0.056	0.020	0.986
White Other	0.020	0.009	0.283	0.435	0.014
Black	0.017	0.011	0.157	0.166	0.000
Asian	0.030	0.012	0.389	0.267	0.000
Mixed	0.021	0.007	0.116	0.113	0.000
Occupational class					
Managerial/Professional	0.791	0.136	0.000	0.402	0.000
Intermediate	0.179	0.332	0.000	0.233	0.000
Routine/manual	0.030	0.532	0.000	0.366	0.000
Not worked recently	0.000	0.000	1.000	0.000	1.000
Employment					
Employed	0.971	0.892	0.000	0.897	0.000
Unemployed	0.016	0.070	0.000	0.077	0.000
Economically inactive	0.013	0.026	0.479	0.026	0.229
Student	0.000	0.012	0.194	0.000	0.018
Retired	0.000	0.000	0.327	0.000	0.753
Education					
No qualification	0.000	0.190	0.282	0.141	0.452
GCSE or equivalent	0.108	0.438	0.242	0.221	0.253
A-level or equivalent	0.125	0.291	0.144	0.116	0.091
Degree level or equivalent	0.767	0.081	0.333	0.522	0.205
Sexual orientation*					
Heterosexual	0.953	0.966	0.936	0.956	0.945
Sexual minority	0.047	0.034	0.064	0.044	0.055
Migrant status					
Non-migrant	0.961	0.988	0.217	0.042	0.989
Migrant	0.039	0.012	0.783	0.958	0.011
Gender					
Male	0.419	0.442	0.315	0.410	0.380
Female	0.581	0.558	0.685	0.590	0.620

*LC1* Employed White British, high education and social class, *LC2* Employed White British, lower education and social class, *LC3* Non-working migrants, varied ethnicity and education, *LC4* Employed migrants, varied ethnicity, social class and education, *LC5* Retired White British

*Unclassified (those aged 65+ who were not asked to disclose their sexual orientation) not displayed here

### Discrimination and social status

Our first and second hypotheses relating to elevated discrimination and MHSU/treatment among disadvantaged social groups and to differential patterns for single versus multiple social status analyses, were partially supported by our findings.

#### Single status modelling

Following adjustments for relevant socio-demographic/economic variables, there were significantly greater odds of past-year discrimination among those aged 16–24 years than older age groups, sexual minority compared to heterosexual participants, ethnic minority (particularly of Black ethnicity) compared to White British participants, migrants versus non-migrants, economically inactive compared to employed people, and those with degree-level educational attainment, compared to those with no qualifications (Table 2).

**Table 2:** Single social statuses and among participants who experienced past-year discrimination (*n* = 542, 7.8%). Unadjusted/ Adjusted Odds Ratios (OR/AOR) and 95% confidence intervals (CI) are shown

	*n*	%	OR	CI	AOR*	CI
Age group (years)						
16–24	89	15.7	1.00		1.00	
25–34	100	9.7	0.58	0.41, 0.81	0.70	0.48, 1.00
35–44	88	7.7	0.45	0.32, 0.63	0.59	0.40, 0.87
45–54	110	7.2	0.41	0.30, 0.58	0.54	0.36, 0.81
55–64	92	6.4	0.37	0.26, 0.52	0.47	0.31, 0.70
65–74	36	2.8	0.16	0.10, 0.24	0.20	0.12, 0.34
75+	27	2.2	0.12	0.07, 0.19	0.13	0.08, 0.23
Gender						
Male	209	7.5	1.00		1.00	
Female	333	8.0	1.08	0.88, 1.33	1.11	0.90, 1.38
Sexual orientation**						
Heterosexual	419	8.7	1.00		1.00	
Sexual minority	60	30.4	4.61	3.22, 6.59	4.07	2.83, 5.86
Ethnicity						
White British	379	6.1	1.00		1.00	
White Other	49	13.6	2.43	1.69, 3.49	2.06	1.42, 2.98
Black	42	20.9	4.08	2.68, 6.20	3.23	2.09, 4.98
Asian	49	14.7	2.66	1.84, 3.83	1.86	1.26, 2.73
Mixed	18	9.9	1.70	0.96, 3.02	1.29	0.69, 2.38
Migration status						
Non-migrant	427	6.8	1.00		1.00	
Migrant	112	13.3	2.10	1.63, 2.72	1.79	1.37, 2.34
Employment status						
Employed	301	8.2	1.00		1.00	
Unemployed	35	14.7	1.93	1.21, 3.07	1.39	0.86, 2.26
Economically Inactive	113	10.3	1.28	0.99, 1.67	1.27	0.97, 1.66
Student	24	16.0	2.13	1.30, 3.49	1.08	0.62, 1.89
Retired	69	2.7	0.31	0.23, 0.41	0.72	0.44, 1.17
Educational attainment						
No qualification	89	4.8	1.00		1.00	
GCSE	139	7.6	1.66	1.20, 2.29	1.17	0.84, 1.64
A-level	94	8.7	1.91	1.33, 2.73	1.13	0.77, 1.66
Degree level	216	9.3	2.05	1.52, 2.77	1.67	1.20, 2.33
Social class						
Managerial/Professional	141	8.6	1.00		1.00	
Intermediate	90	8.5	0.99	0.72, 1.36	0.95	0.68, 1.31
Routine/manual	104	8.1	0.93	0.69, 1.27	0.71	0.52, 0.98
Not worked recently	166	5.4	0.60	0.46, 0.78	1.13	0.83, 1.55

*Adjusted for age, marital status, urban/rural residence, area deprivation

**Unclassified (those aged 65+ who were not asked to disclose their sexual orientation) not displayed here

#### Intersectional analysis modelling

Intersectional analyses indicated that, after adjusting for additional socio-demographic confounders not included in the LCA models (age, marital status, urban/rural residence and area-level deprivation), the ‘Employed migrant’ (AOR 2.14, CI 1.57–2.92) and ‘Retired White British’ (AOR 1.37, CI 1.04–1.81) groups had greater odds of reporting past-year discrimination compared to the most privileged group (Table 3).

**Table 3:** Intersectional social statuses and past-year discrimination. Unadjusted/Adjusted Odds Ratios (OR/AOR) and 95% confidence intervals (CI) are shown

Latent Classes (LC)	Unadjusted OR	CI	AOR*	CI
LC 1 (*n* = 1791)	1.00		1.00	
LC 2 (*n* = 1994)	0.97	0.75, 1.24	0.80	0.80, 0.62
LC 3 (*n* = 348)	1.52	1.03, 2.25	1.31	1.31, 0.87
LC 4 (*n* = 524)	2.38	1.77, 3.21	2.14	2.14, 1.57
LC 5 (*n* = 2889)	0.80	0.63, 1.01	1.37	1.37, 1.04

*LC1* Employed White British, high education and social class, *LC2* Employed White British, lower education and social class, *LC3* Non-working migrants, varied ethnicity and education, *LC4* Employed migrants, varied ethnicity, social class and education, *LC5* Retired White British

*Adjusted for age, marital status, urban/rural residence, area deprivation

### Mental health service use/treatment and social status

As illustrated in Table 4, all markers of need and past-year discrimination were positively associated with MHSU/treatment.

**Table 4:** Associations between single social statuses and mental health service use (MHSU)/treatment

	Any MHSU/treatment		Unadjusted OR		AOR 1		AOR 2		AOR3		AOR4	
	*n*	%	OR	CI	OR	CI	OR	CI	OR	CI	OR	CI
Age category												
16–24 (*N* = 560)	91	13.8	1.00		1.00		1.00		1.00		1.00	
25–34 (*N* = 1035)	212	17.7	1.34	0.99, 1.82	1.30	0.89, 1.9	1.33	0.86, 2.04	1.31	0.85, 2.02	1.35	0.88, 2.09
35–44 (*N* = 1180)	269	20.0	1.56	1.17, 2.09	1.59	1.09, 2.31	1.61	1.06, 2.44	1.50	0.99, 2.28	1.58	1.03, 2.41
45—54 (*N* = 1294)	311	21.5	1.71	1.28, 2.27	1.66	1.15, 2.42	1.69	1.12, 2.56	1.39	0.91, 2.13	1.47	0.96, 2.25
55–64 (*N* = 1226)	306	23.2	1.89	1.42, 2.51	1.71	1.16, 2.52	1.85	1.19, 2.86	1.42	0.91, 2.22	1.50	0.95, 2.35
65–74 (*N* = 1189)	211	17.0	1.28	0.96, 1.72	1.18	0.65, 2.15	1.73	0.86, 3.47	0.96	0.47, 1.94	0.97	0.48, 1.96
75+ (*N* = 1062)	176	15.9	1.18	0.87, 1.59	0.92	0.49, 1.71	1.46	0.71, 3.01	0.77	0.37, 1.6	0.79	0.38, 1.63
Gender												
Male (*N* = 3058)	464	13.7	1.00		1.00		1.00		1.00		1.00	
Female (*N* = 4488)	1112	23.5	1.93	1.69, 2.22	1.74	1.5, 2.02	1.60	1.37, 1.88	1.71	1.45, 2.02	1.73	1.47, 2.04
Sexual orientation												
Heterosexual (*N* = 4834)	1047	18.4	1.00		1.00		1.00		1.00		1.00	
Not heterosexual (*N* = 223)	74	32.7	2.15	1.54, 2.99	2.23	1.52, 3.27	1.75	1.16, 2.64	1.65	1.09, 2.5	1.50	0.99, 2.28
N/A (*N* = 2489)	455	17.5	0.94	0.82, 1.08	1.06	0.72, 1.57	0.92	0.57, 1.48	1.34	0.84, 2.16	1.40	0.87, 2.25
Ethnicity												
White British (*N* = 6387)	1389	20.0	1.00		1.00		1.00		1.00		1.00	
White Other (*N* = 426)	66	12.7	0.58	0.42, 0.8	0.83	0.52, 1.3	0.88	0.55, 1.43	0.88	0.53, 1.44	0.83	0.5, 1.38
Black (*N* = 197)	27	10.4	0.46	0.3, 0.73	0.48	0.28, 0.8	0.38	0.21, 0.66	0.36	0.2, 0.66	0.32	0.17, 0.6
Asian (*N* = 357)	52	13.3	0.62	0.43, 0.88	0.70	0.41, 1.2	0.66	0.37, 1.18	0.67	0.36, 1.23	0.63	0.34, 1.17
Mixed (*N* = 151)	31	16.8	0.81	0.52, 1.27	0.95	0.55, 1.65	0.88	0.49, 1.61	0.87	0.47, 1.6	0.83	0.46, 1.52
Migration status												
Non-migrant (*N* = 6601)	1432	19.8	1.00		1.00		1.00		1.00		1.00	
Migrant (*N* = 914)	134	12.5	0.58	0.46, 0.72	0.66	0.45, 0.98	0.66	0.43, 1	0.68	0.44, 1.05	0.69	0.45, 1.06
Employment status												
Employed (*N* = 3996)	659	14.8	1.00		1.00		1.00		1.00		1.00	
Unemployed (*N* = 218)	56	20.3	1.47	1.02, 2.11	1.73	1.17, 2.55	1.22	0.79, 1.88	1.13	0.73, 1.75	1.12	0.73, 1.73
Economically inactive (*N* = 919)	422	43.0	4.36	3.65, 5.19	2.43	1.33, 4.44	2.10	1.07, 4.11	2.02	1.05, 3.9	2.02	1.03, 3.96
Student (*N* = 146)	24	12.6	0.83	0.5, 1.39	1.52	0.5, 4.66	1.68	0.5, 5.64	1.79	0.52, 6.2	1.65	0.46, 5.93
Retired (*N* = 2267)	415	17.4	1.22	1.05, 1.42	0.78	0.39, 1.53	1.02	0.48, 2.19	1.13	0.53, 2.41	1.13	0.53, 2.45
Educational attainment												
No qualification (*N* = 1843)	449	23.2	1.00		1.00		1.00		1.00		1.00	
GCSE (*N* = 2019)	434	19.5	0.80	0.68, 0.96	0.99	0.81, 1.21	1.01	0.81, 1.26	1.07	0.86, 1.35	1.07	0.85, 1.35
A-level (*N* = 1192)	241	17.3	0.69	0.57, 0.85	1.01	0.79, 1.29	1.08	0.83, 1.41	1.14	0.87, 1.49	1.13	0.86, 1.48
Degree level (*N* = 2410)	431	15.9	0.63	0.53, 0.74	0.89	0.71, 1.11	1.01	0.8, 1.28	1.06	0.83, 1.36	1.04	0.81, 1.33
Social class												
Managerial/Professional (*N* = 1795)	296	15.1	1.00		1.00		1.00		1.00		1.00	
Intermediate (*N* = 1104)	203	16.9	1.14	0.91, 1.43	0.99	0.78, 1.25	0.97	0.75, 1.25	0.99	0.77, 1.29	0.99	0.77, 1.28
Routine/manual (*N* = 1342)	235	14.5	0.95	0.77, 1.18	0.83	0.65, 1.06	0.82	0.63, 1.07	0.84	0.64, 1.11	0.84	0.64, 1.1
Not worked recently (*N* = 2942)	751	25.6	1.94	1.64, 2.29	1.58	0.84, 2.96	1.16	0.58, 2.32	1.02	0.51, 2.02	1.02	0.51, 2.05
Index of multiple deprivation quintiles												
1 (least) (*N* = 1554)	1554	13.4			1.00		1.00		1.00		1.00	
2 (*N* = 1550)	1550	16.7			1.31	1.04, 1.64	1.23	0.97, 1.57	1.21	0.95, 1.55	1.21	0.95, 1.54
3 (*N* = 1563)	1563	18.7			1.44	1.15, 1.8	1.25	0.98, 1.58	1.19	0.94, 1.52	1.19	0.94, 1.51
4 (*N* = 1457)	1457	20.3			1.58	1.25, 2	1.32	1.03, 1.7	1.26	0.97, 1.63	1.26	0.97, 1.62
5 (most) (*N* = 1422)	1422	24.2			1.86	1.46, 2.37	1.44	1.1, 1.89	1.33	1.02, 1.74	1.35	1.03, 1.77
Discrimination												
None (*N* = 7004)	1353	17.3					1	1.00, 1.00	1	1.00, 1.00	1	1.00, 1.00
Any (*N* = 542)	223	35.4					3.17	2.48, 4.06	1.85	1.39, 2.44	1.60	1.20, 2.12
CIS-R Score												
0–5 (*N* = 5097)	523	8.8							1	1.00, 1.00	1	1.00, 1.00
6–11 (*N* = 1213)	322	24.8							2.99	2.47, 3.62	2.32	1.90, 2.84
12–17 (*N* = 580)	279	44.7							6.75	5.37, 8.49	4.82	3.78, 6.14
18 (*N* = 656)	452	65.8							14.67	11.46, 18.78	9.29	7.13, 12.09
Self-rated overall health												
Excellent/v good/good (*N* = 5890)	896	13.7									1	1.00, 1.00
Fair/poor (*N* = 1656)	680	40.6									1.90	1.56, 2.31
Physical illness												
None (*N* = 2037)	230	9.6									1	1.00, 1.00
Any (*N* = 5509)	1346	23.0									1.69	1.37, 2.10
Social functioning												
Good (*N* = 3673)	476	11.3									1	1.00, 1.00
Poor (*N* = 3873)	1100	25.3									1.66	1.39, 1.98

Numbers (*n*), weighted percentages (%), Unadjusted/Adjusted Odds Ratios (OR/AOR) and 95% confidence intervals (CI) are shown—footnotes specify variables adjusted for

CIS-R = Revised Clinical Interview Schedule; Economically inactive here refers to volunteers, those on maternity leave, fulltime carers, fulltime parents and those who are unable to work due to their health

^1^Adjusted for age, gender, marital status, urban/rural residence, ethnicity, social class, employment status, migration status, sexual orientation, educational attainment, area deprivation

^2^As 1, additionally adjusted for any past-year discrimination

^3^As 2, additionally adjusted for grouped CIS-R score

^4^As 3, additionally adjusted for self-reported general health, physical illness and social functioning

#### Single status modelling

Following adjustments for socio-demographic/economic variables, additional adjustments for need fully or partially attenuated positive associations between MHSU/treatment and minority sexual orientation, migrant status, being unemployed and greater deprivation. There remained elevated MHSU/treatment among females compared to males (AOR 1.71, CI 1.45–2.02), those who were economically inactive (AOR 2.02, CI 1.05–3.90), sexual minority (AOR 1.65, CI 1.09–2.50), and those living in the most deprived quintile (AOR 1.33, CI 1.02–1.74) compared to the most advantaged group. Those identifying as any Black ethnic group had lower odds of MHSU/treatment compared to White British participants, and adjustments for need strengthened rather than attenuated this negative association (AOR 0.36, CI 0.20–0.66).

#### Intersectional analysis modelling

Using the typology generated by latent class analyses (Table 5) in models adjusted for remaining socio-demographic/economic variables, compared to the most privileged White British group, the ‘Non-working migrants’ and ‘Retired White British’ had greater odds of MHSU/treatment, while the ‘Employed migrants’ had lower odds. Adjustments for need accounted for the association for ‘Non-working migrants’ (fully accounted for by common mental disorder symptoms), and partially accounted for the association for the ‘Retired White British’ group, though this remained significant (AOR 1.88, CI 1.53–2.32). Adjustments for need had little impact on the negative association with MHSU/treatment observed for ‘Employed migrants’ (AOR 0.39, CI 0.27–0.55).

**Table 5:** Associations between intersectional latent class membership and mental health service use (MHSU)/treatment

	Unadjusted OR		Adjusted OR 1		Adjusted OR 2		Adjusted OR 3		Adjusted OR 4	
	OR	CI	OR	CI	OR	CI	OR	CI	OR	CI
LC 1 (*n* = 1791)										
LC 2 (*n* = 1994)	1.20	1.01, 1.41	1.14	0.96, 1.36	1.17	0.98, 1.39	1.09	0.90, 1.32	1.09	0.90, 1.32
LC 3 (*n* = 348)	1.60	1.22, 2.11	1.78	1.33, 2.37	1.73	1.29, 2.32	1.32	0.96, 1.83	1.30	0.93, 1.82
LC 4 (*n* = 524)	0.49	0.36, 0.68	0.44	0.31, 0.60	0.39	0.28, 0.54	0.36	0.25, 0.52	0.39	0.27, 0.55
LC 5 (*n* = 2889)	1.71	1.47, 1.98	3.00	2.50, 3.61	2.98	2.47, 3.58	2.06	1.68, 2.53	1.88	1.53, 2.32

Reference is the designated most privileged class. Unadjusted and Adjusted Odds Ratios (OR/AOR) and 95% confidence intervals (CI) are shown

*LC1* Employed White British, high education and social class, *LC2* Employed White British, lower education and social class, *LC3* Non-working migrants, varied ethnicity and education, *LC4* Employed migrants, varied ethnicity, social class and education, *LC5* Retired White British

^1^Adjusted for age, marital status, urban/rural residence, area deprivation

^2^As 1, additionally adjusted for any past-year discrimination

^3^As 2, additionally adjusted for grouped CIS-R score

^4^As 3, additionally adjusted for self-reported general health, physical illness and social functioning

### Discrimination and mental health service use/treatment

Our third and fourth hypotheses, that accounting for past-year discrimination would attenuate associations with MHSU/treatment, and that the influence of discrimination on MHSU/treatment would be greater for people with multiple disadvantaged social statuses, were in general not supported by our findings.

#### Single status modelling

Adjustments for discrimination had little or no influence on effect sizes for most single status groups (Table 4). However, for sexual minorities relative to heterosexuals, the association became non-significant after adjustment (AOR 1.50, CI 0.99–2.28), indicating discrimination partly accounted for some of the elevated MHSU/treatment in this group. In contrast, a positive association with MHSU/treatment emerged for those aged 35–44 years compared to the youngest age group (AOR 1.58, CI 1.03–2.41).

#### Intersectional analysis modelling

In intersectional analyses, adjustments for discrimination had no influence on respectively positive and negative associations with MHSU/treatment for the ‘Retired White British’ and ‘Employed migrants’ groups despite finding significantly greater odds of discrimination in these same two groups (Tables 3, 5).

## Discussion

Using population-level data, we examined experiences of past-year discrimination, mental health service use (MHSU) and treatment. Controlling for need, we compared patterns across single and intersectional social statuses (using latent class analysis) and examined the influence of discrimination on MHSU/treatment. Analyses revealed different patterns of discrimination and MHSU/treatment when examining single or intersectional social groups. Single status analyses identified characteristics (e.g., being a sexual minority, Black or female) associated with MHSU/treatment (and discrimination) that were not distinctive markers of intersecting class membership in the data-driven models, and therefore were not highlighted by our intersectional approach. Intersectional analyses also detected patterns not observed by single statuses, finding the “Retired White British” group had greater odds and “Employed migrants” lower odds of MHSU/treatment following adjustments for need. Though discrimination was associated with certain social statuses and with greater MHSU/treatment, there was little evidence that it acted as a mediator in either analytic approach.

### Examining single versus multiple social statuses

Our findings support the importance of multiple approaches to understanding complexities of social stratification and support prior research identifying differences in mental health and healthcare at the junctures of different social identities [21, 59]. While the variables included in LCA analyses were theoretically considered in relation to salient social stratification categorisations, the data-driven approach allows examination of classes which commonly occur in the population of interest (and are thus sensitive to context). This may mask important inequalities among less frequent classes within the populations and/or who are underrepresented in surveys (e.g., UK-born ethnic minorities). Complimentary analyses could examine a priori defined dyads or triads of social status depending on the research focus.

There are other ways of tailoring analyses to the research question or conceptualisation of intersectionality. For instance, in using LCA, our study took an ‘intercategorical’ approach, looking at dimensions across categories of social status [35]. Therefore, individuals classified, for example, as ‘non-working migrants’ were placed at the intersection of employment and migration status, but in a way that reduces these heterogeneous experiences into a single dimension for measurement. While this may be helpful for public health professionals to have a more nuanced picture of inequities in the population, those interested in understanding the experiences of people occupying particular intersections of social status (perhaps selected on the basis of intercategorical findings) may prefer to conduct ‘intracategorical’ analyses, which lend themselves more to qualitative approaches [35]. Nonetheless, it is important to move beyond describing patterns and emphasise understanding how the experience of intersecting identities might affect mental health and service use. This paper has been able to achieve this by not just using LCA to identify homogeneous patterns of intersectionality that occur within the data, but also examine how people with similar intersectional profiles engage with health services. Future work can build on this by taking a more targeting approach, possibly with the use of coded intersections and/or multilevel modelling to establish whether specific combinations of characteristics drive inequalities more than others.

### Social status and MHSU/treatment

After accounting for need, MHSU/treatment was elevated among females, sexual minorities, economically inactive and participants living in the most deprived areas in single status analyses, but lower among Black participants. This reflects previously reported findings [39] and the wider literature (e.g., [54]. Our study extends this to indicate how MHSU/treatment among intersectional social groups common in the population compare to the most privileged (multiply advantaged) group.

MHSU/treatment was elevated for the ‘Retired White British’ latent class but was significantly lower for the ‘Employed migrants’ group. Being retired was not associated with MHSU/treatment in single status analyses, thus our intersectional findings for this class were not expected. This latent class was the largest, reflecting that White British make up the majority of the population in England and retirement is proportionately more common in this group given its older age profile compared to other racial/ethnic groups (ONS 2011).

#### Residual confounding by need

Elevated MHSU/treatment may be partly due to residual confounding by mental health: our measure of common mental disorders reflects current mental health (in the past two weeks) while MHSU/treatment referred to the past year. Therefore, given the relapsing–remitting nature of symptoms of anxiety and depression, it may not adequately capture people’s mental health during that time. However, this caveat would have also held for other intersectional groups with initially elevated MHSU/treatment (the ‘Non-working migrants’ group), for whom adjustments for mental health completely attenuated the association.

Similarly, although the lower odds of MHSU/treatment in the ‘Employed migrants’ group remained despite adjustments for differences in age and need, there may be some residual confounding by need reflective of a ‘healthy migrant effect’ that would not necessarily have been observed for the ‘Non-working migrants’ group, which had greater levels of economic activity.

#### Opportunity costs

Greater MHSU/treatment among the ‘Retired White British’ group may also reflect lower opportunity costs (e.g., lack of work commitments, greater leisure time) associated with seeking help, as has been reported previously for healthcare utilisation among retirees more generally [66]. Opportunity costs may also have influenced lower MHSU/treatment among the ‘Employed migrants’ group (comprising predominantly White Other and to a lesser extent Asian ethnic groups). This group had a greater conditional probability of having degree-level education than the retired or lower social class White British group, yet, it was heterogeneous in terms of occupational social class. This reflects UK statistics that migrants are more likely to experience downward social mobility, working in jobs for which they are overqualified, which is associated with poorer mental health [15]. This is particularly the case for those from recent European Union (EU) accession countries (included in the White Other category) who are more likely to be in low-skilled occupations than migrants from India, East, and Southeast Asia (Migration [41] and more likely to have non-standard work (e.g., temporary/short/part-time/daily work) [19]. Such occupational circumstances may be less flexible and accrue greater opportunity costs to seeking healthcare [62].

This explanation is undermined by the discrepancy found between the ‘Employed White British, low social class’ and the ‘Employed migrants’ group. The lower social class White British group had a greater probability of routine/manual skilled work (potentially less flexible, with higher opportunity costs of help-seeking) and lower education than the employed migrants group; yet, they did not have significantly different odds of MHSU/treatment. However, in support of the opportunity cost explanation, the ‘Employed migrants’ group comprised a mix of occupational social classes with a greater conditional probability of being managerial/professional than the lower social class employed White British group. Further, this ‘Employed migrants’ group may more commonly comprise self-employed people [12], accruing greater opportunity costs in terms of seeking mental health care than those working in more standard employment, thus partially explaining lower MHSU/treatment.

#### Unmeasured confounders: social integration and networks

Finally, length of residence may be an important unmeasured confounder for the migrant groups. Compared to the ‘Non-working migrants’ group, the ‘Employed migrants’ group is likely to include a greater proportion of more recently arrived migrants [20]. Previous UK evidence indicates that more recent (but not longer-resident) migrants had lower odds of being registered with a GP, particularly White migrants and those who migrated for work [20]. Lower MHSU/treatment in the employed migrant group may thus reflect lack of integration into the healthcare system and lack of access to treatment—particularly since this is predominantly gatekept by primary care. They may also be less familiar with services available which could limit opportunities for self-referral, or have concerns about seeking out help which act as a barrier to care. For example, Polling et al. [51] found that (among an ethnically diverse UK sample), often those who require support or health services do not seek help because they anticipated being treated unfairly, cultural insensitivity of care or concerns that they will be brought to the attention of other authorities.

### Influence of discrimination on mental health service use

As with MHSU/treatment, observed patterns of discrimination differ when considering social status singly or intersectionally. Past-year discrimination was positively associated with specific disadvantaged single social statuses and was significantly elevated in the two intersectional classes associated MHSU/treatment (‘Retired White British’ and ‘Employed migrants’), but not—as might be expected—in the multiply disadvantaged ‘Non-working migrants’ group. Previous research has identified higher odds of discrimination among White Other compared to White British groups, particularly for more recent migrants [26], though the findings for the ‘Retired White British’ group were less expected. While we did not examine attributions, it is possible that discrimination on the basis of age was more common in this latter group.

However, while discrimination predicted greater MHSU/treatment, adjustments for discrimination had little or no influence on associations MHSU/treatment in either single (except sexual orientation) or intersectional status analyses. This contrasts with previous findings [21, 24] though these studies were able to examine different forms of discrimination which may explain the discrepancy. For example, anticipated, but not everyday or major experiences of discrimination was associated with increased MHSU/treatment in analyses adjusting for intersectional social status and need [21]. Also, while a recent review indicates that racism predicts poorer satisfaction, reduced trust, and possibly delayed access to services, it may not predict health service use [7]. Further work may therefore usefully examine whether discrimination has differential effects on different aspects of service use (e.g., delayed treatment, adherence, treatment pathways) for multiply disadvantaged groups. Finally, only a small proportion of APMS participants disclosed their experiences of discrimination. Though underreporting may not be unexpected in such surveys, there may be differences in likelihood to disclose different discrimination types (e.g., ageism, sexism, racism).

Additional qualitative or policy development work to improve MHSU could be achieved is by identifying people that have key intersecting characteristics defined by the model. For example, recruiting heterosexual, White British people who are educated to degree level and are employed in senior roles would reflect latent class 1, while recruiting economically inactive/retired migrants from a variety of ethnic minority groups would reflect latent class 3.

### Strengths and limitations

Key strengths of this study are its use of national-level data representative of households in England building on an approach previously used in a regional UK sample. Also, our approach to identifying inequalities in MHSU/treatment moves beyond single status analyses to incorporate an intersectional approach using LCA which identifies common intersectional classes in the population and picks up patterns not visible in single status analyses alone—though LCA can sometimes focus on certain categories and ignore others, which is a limitation of quantitative explorations of intersectionality [16]. Our approach is one approach to examining intersectionality, specifically identifying latent classes present in the population in terms of probability of belonging to potentially marginalised social statuses and contrasting exposure to discrimination (a form of oppression) outcomes across groups classified as multiply advantaged, disadvantaged or a mix of advantaged and disadvantaged, and thus with differing positions in terms of social power, and differing ‘vulnerability’ to health inequalities (e.g., [36]. We agree that this differs from examining the experiences of individuals/groups at the intersections of two or more social statuses but consider our approach to add (although partially and incrementally) to our understanding of how experiences are distributed in the population beyond examining single social statuses at a time. A further strength is that we move beyond describing inequities to examine discrimination as a potential underpinning mechanism driving such inequalities.

The main limitation of this study is our use of a binary measure of discrimination. This was necessary due to low n and the limited nature of the discrimination measures in APMS, though this does underestimates the complexity of experienced discrimination in patterning health outcomes. Similarly, it was also necessary to dichotomise sexual orientation due to low n, potentially obscuring findings from specific orientation. APMS is also a survey of private households, thus excluding individuals living in institutions or who are homeless (though there are small groups of APMS participants who have experienced homelessness) who may be more likely to experience poor mental health and less likely to receive help. Furthermore, as data were cross-sectional, mediation by discrimination could not have been causally inferred due to the concurrent temporal reporting. Also, although this is a national sample, participant numbers in some racial/ethnic minority groups were small. This may have impacted on sample size/inferences, exemplifying the need for surveys to implement ethnic minority booster samples. Finally, although a pervasive adversity, the measures of discrimination used here did not directly enquire about fear of discrimination from mental health services, and thus may not have adequately captured underpinning mechanisms relevant to MHSU/treatment.

## Conclusion

Patterns of both MHSU/treatment and discrimination differ when considering single and multiple statuses, highlighting how both approaches each identify inequities that may not otherwise have been apparent. There is little evidence that our measure of discrimination affects associations between social status and MHSU/treatment. However, to improve understanding and inform action on social status-based inequalities, further research should incorporate multiple and mixed-methods approaches to identify and characterise the complexities of social stratification processes, examine different dimensions of inequalities in mental health problems and care, different health service delivery contexts, as well as different and more specific aspects of discrimination.

## Supplementary Information

Below is the link to the electronic supplementary material.

Supplementary file1 (DOCX 1616 KB)
