# Supplementary material for: A comparison of single and intersectional social identities associated with discrimination and mental health service use: data from the 2014 Adult Psychiatric Morbidity Survey in England
Source: Soc Psychiatry Psychiatr Epidemiol. 2022 Mar 7;57(10):2049–63. doi: 10.1007/s00127-022-02259-1 (PMC9477952; doi:10.1007/s00127-022-02259-1)
Supplement: Supplementary file 1 — Supplementary file1 (DOCX 1616 KB) [file 127_2022_2259_MOESM1_ESM.docx]

**Supplementary File - Goodness of fit statistics**

 The supplementary Tables below report the goodness of fit statistics for models with two – seven classes for both male and female participants. As well as entropy and log likelihood, Lo-Mendell-Rubin adjusted (LMRA), Akaike information criterion (AIC) and Sample Size Adjusted Bayesian Information Criterion (SSABIC) are reported. The most appropriate model with the best fit statistics is highlighted in Table S1 and Table S2.

*Table S1: Goodness of fit statistics for male participants (n=3058)*

| **Number of classes** | **Entropy** | **Log likelihood** | **LMRA** | **AIC** | **SSABIC** |
| --- | --- | --- | --- | --- | --- |
| 2 | 0.982 | -12504.271 | 0.0000 | 25074.543 | 25168.530 |
| 3 | 0.971 | -12051.802 | 0.0000 | 24203.604 | 24346.010 |
| 4 | 0.882 | -11823.365 | 0.0000 | 23780.730 | 23971.554 |
| 5 | 0.900 | -11667.194 | 0.0000 | 23502.388 | 23741.630 |
| 6 | 0.906 | -11621.035 | 0.0166 | 23444.070 | 23731.729 |
| 7 | 0.914 | -11597.004 | 0.2127 | 23430.008 | 23766.086 |

*Table S2: Goodness of fit statistics for female participants (n=4488)*

| **Number of classes** | **Entropy** | **Log likelihood** | **LMRA** | **AIC** | **SSABIC** |
| --- | --- | --- | --- | --- | --- |
| 2 | 0.982 | -18663.336 | 0.0000 | 37392.672 | 37499.313 |
| 3 | 0.980 | -17963.029 | 0.0000 | 36026.059 | 36187.636 |
| 4 | 0.915 | -17649.833 | 0.0000 | 35433.666 | 35650.180 |
| 5 | 0.902 | -17350.515 | 0.0000 | 34869.030 | 35140.481 |
| 6 | 0.890 | -17261.935 | 0.0000 | 34725.870 | 35052.257 |
|  | 0.890 | -17213.602 | 0.7765 | 34663.203 | 35044.527 |
